# A Predictive Model for Assessing Surgery-Related Acute Kidney Injury Risk in Hypertensive Patients: A Retrospective Cohort Study

**DOI:** 10.1371/journal.pone.0165280

**Published:** 2016-11-01

**Authors:** Xing Liu, Yongkai Ye, Qi Mi, Wei Huang, Ting He, Pin Huang, Nana Xu, Qiaoyu Wu, Anli Wang, Ying Li, Hong Yuan

**Affiliations:** 1 Department of Cardiology, The Third Xiangya Hospital, Central South University, Changsha, 410013, The People’s Republic of China; 2 School of Computer, National University of Defense Technology, Changsha, 410073, The People’s Republic of China; 3 Department of Sports Medicine and Nutrition, University of Pittsburgh, PA, 15260, United States of America; 4 Center of Clinical Pharmacology, The Third Xiangya Hospital, Central South University, Changsha, 410013, The People’s Republic of China; 5 Information Department, The Third Xiangya Hospital, Central South University, Changsha, 410013, The People’s Republic of China; Emory University Department of Medicine, UNITED STATES

## Abstract

**Background:**

Acute kidney injury (AKI) is a serious post-surgery complication; however, few preoperative risk models for AKI have been developed for hypertensive patients undergoing general surgery. Thus, in this study involving a large Chinese cohort, we developed and validated a risk model for surgery-related AKI using preoperative risk factors.

**Methods and Findings:**

This retrospective cohort study included 24,451 hypertensive patients aged ≥18 years who underwent general surgery between 2007 and 2015. The endpoints for AKI classification utilized by the KDIGO (Kidney Disease: Improving Global Outcomes) system were assessed. The most discriminative predictor was selected using Fisher scores and was subsequently used to construct a stepwise multivariate logistic regression model, whose performance was evaluated via comparisons with models used in other published works using the net reclassification index (NRI) and integrated discrimination improvement (IDI) index.

**Results:**

Surgery-related AKI developed in 1994 hospitalized patients (8.2%). The predictors identified by our Xiang-ya Model were age, gender, eGFR, NLR, pulmonary infection, prothrombin time, thrombin time, hemoglobin, uric acid, serum potassium, serum albumin, total cholesterol, and aspartate amino transferase. The area under the receiver-operating characteristic curve (AUC) for the validation set and cross validation set were 0.87 (95% CI 0.86–0.89) and (0.89; 95% CI 0.88–0.90), respectively, and was therefore similar to the AUC for the training set (0.89; 95% CI 0.88–0.90). The optimal cutoff value was 0.09. Our model outperformed that developed by Kate et al., which exhibited an NRI of 31.38% (95% CI 25.7%-37.1%) and an IDI of 8% (95% CI 5.52%-10.50%) for patients who underwent cardiac surgery (n = 2101).

**Conclusions/Significance:**

We developed an AKI risk model based on preoperative risk factors and biomarkers that demonstrated good performance when predicting events in a large cohort of hypertensive patients who underwent general surgery.

## Introduction

Acute kidney injury (AKI) is a serious post-surgery complication and a strong predictor of various long-term adverse outcomes [1–6], including chronic kidney disease (CKD), end-stage renal disease (ESRD), cardiovascular disease (heart failure and myocardial infarction), and increased mortality (12.32% of AKI patients die in the hospital despite appropriate treatment, and 35% of AKI patients die within one year of injury) [1,7]. Preventative measures have been implemented to improve patient outcomes; however, AKI remains a frequent post-surgery complication, with rates ranging from 3% to 43% across various studies [2,3,8,9].

Hypertension is a worldwide public health problem affecting billions of people of all races and ethnicities. According to the 2015 Report on the Status of Nutrition and Chronic Diseases in China [10], approximately 25.2% of adults suffer from hypertension, which is estimated to affect 270 million people nationwide. Patients with hypertension are at increased risk for AKI and often require early and intensive care [9,11]. However, no pharmacotherapies for treating AKI in adults exist, and only limited methods for preventing AKI are available [12]. Thus, it is essential to identify and evaluate vulnerable patients—such as patients with hypertension—before these patients undergo surgery.

A few risk prediction models have been used to identify high-risk individuals and thus improve patient prognoses through aggressive interventions; however, no preoperative risk models for AKI have been developed for hypertensive patients undergoing general surgery. Thus, in this study, we analyzed preoperative risk factors in a large cohort of hypertensive patients and developed and validated a risk model for surgery-related AKI.

## Methods

### Data Source

We conducted a single-center, retrospective cohort study of hospitalized adults. All de-identified data were obtained from the structured hospital information system (HIS) of the 3^rd^ Xiangya Hospital (Changsha, China), which serves as the sole portal for clinical data entry for all public inpatient settings across different regions of China and provides the complete patient health record information, including information regarding age, sex, race, body mass index (BMI), blood pressure (BP), hospital service type (medical, surgical, or other), International Classification of Disease (ICD-10) clinical diagnosis, surgery date, surgical sites, and laboratory data. Information regarding comorbidities, including cardiovascular risk factors and medical conditions, such as “pulmonary infection”, “chronic kidney disease”, “diabetes” or “impaired glucose tolerance”, “malignant tumor”, “viral hepatitis”, and “heart failure”, was extracted from the system using ICD-10 codes. The study protocol conformed to the ethical guidelines of the 1975 Declaration of Helsinki, and the provision of informed consent was not necessary because all patients were anonymized. The Institutional Review Board of Third Xiangya Hospital approved this study (S1 Text, No. 2016-S149), the reporting of which conforms to the Transparent Reporting Guidelines for a Multivariable Prediction Model for Individual Prognosis Or Diagnosis (TRIPOD) [13].

### Patients

Patients who underwent only one elective surgery during hospitalization and had a discharge diagnosis of “hypertension” between October 2007 and June 2015 were considered potential candidates (n = 29,538) for inclusion in this study. All candidates were adults (aged ≥18 years). Patients with missing data regarding age and pre- or postoperative blood creatinine levels (n = 5087) were excluded. Thus, 24,451 eligible patients were randomly classified into either a derivation dataset (70%, n = 17089) or a validation dataset (30%, n = 7362).

### Outcome Variables and Covariates

All included patients were followed up until the development of surgery-related AKI was confirmed based on the KDIGO (Kidney Disease: Improving Global Outcomes) AKI classification system [12] (a serum creatinine increase greater than 0.3 mg/dL within 48 hours or a 1.5-fold increase in serum creatinine within seven days after surgery) using the peak-to-nadir serum creatinine difference. Briefly, the serum creatinine nadir was defined as the lowest value recorded during the first 7 days of hospitalization, and the serum creatinine peak was defined as the highest value recorded during the first 7 days of hospitalization.

The estimated glomerular filtration rate (eGFR) was derived from the serum creatinine nadir recorded during hospitalization using the Chronic Kidney Disease Epidemiology Collaboration (CKD-EPI) creatinine equation [14]. Additional covariates of interest recorded during hospitalization were selected according to reported AKI risk factors [2,9], such as age, gender, average preoperative blood pressure, plasma biomedical markers (routine blood tests, glucose and blood lipids), past medical history (acute coronary heart disease, acute stroke, or acute cerebral hemorrhage within the last six months), personal history (smoking history or alcohol consumption history), and final diagnoses (diabetes mellitus, impaired glucose tolerance, pulmonary infection, malignant tumor, viral hepatitis, or heart failure), as well as vasoactive agent (e.g., norepinephrine, epinephrine, dopamine, metaraminol, isoproterenol, phentolamine, or nitroprusside), contrast medium (e.g., iohexol or iodixanol), lipid-lowering drug, antiplatelet drug (aspirin), anticoagulant drug (e.g., warfarin, clopidogrel, or heparin), or anti-arrhythmic drug (e.g., amiodarone, lidocaine, propafenone, quinidine, or mexiletine) use and surgery sites. These data had a high rate of integrity, as less than 15% of items per variable were missing. Missing data were imputed using an expectation maximization algorithm.

### Statistical Analysis

All covariates were included to compute Fisher scores to identify prognostic factors, and less significant clinical or statistical variables were subsequently excluded one by one during the model-building process. Several candidate models were developed thereafter and compared with respect to their ability to predict AKI using stepwise multivariate logistic regression. Variation inflation factors (VIF) were computed to detect multicollinearity between selected variables, and multicollinearity was determined to be present in cases in which these factors were ≥5. Variables could be evaluated on the basis of subject knowledge. The likelihood-ratio chi-square test was used to examine the linearity assumption for the predictors used in the multivariable model. Akaike information criterion (AIC) values were also determined after fitting several candidate markers. Over-fitting was assessed using cross validation error and training error, although over-fitting may not have been present in this large cohort. A model was considered over-fitted if the training error was far greater than the validation error, and the value of the training error was small.

Sensitivity, specificity, Youden's index, G_mean_, receiver operating characteristic (ROC) curves, and areas under the ROC curve (AUC) were calculated to evaluate the performance of the model developed based on the imbalanced data. In a two-class problem, the confusion matrix has two rows and two columns specifying the number of false positives (FPs), false negatives (FNs), true positives (TPs), and true negatives (TNs), and the above measures are defined as follows: Sensitivity = *TP*/(*TP* + *FN*), Specificity = *TN*/(*TN* + *FP*), Youden index = *Sensitivit* + *Specificity −* 1, and ${\text{G}}_{\text{mean}}\;=\;\sqrt{\frac{TP}{TP+FN}\times \frac{TN}{TN+FP}}\;$. The larger the AUC, the higher the performance of the model. The optimal cutoff value of the model was determined by maximizing the sum of its sensitivity and specificity, and the predictive accuracy of the model was verified via five-fold cross validation.

Predicted probabilities were calculated using published equations, and the net reclassification index (NRI) and integrated discrimination improvement (IDI) were estimated to compare the performances of the new models to those of previously published models. A systematic literature search of Medline (1950-) was performed in February 2016 by professional document retrieval personnel from the Xiang-Ya Medical Library of Central South University using the following search terms: ‘predict’, ‘forecast’, ‘surgery’, ‘operation’, ‘forecasting’, ‘acute kidney injury’, and ‘Surgical Procedures, Operative’, and the reference lists of relevant papers were subsequently reviewed. There were no language restrictions. Studies were included if they utilized a model for predicting adult (≥18 years old) AKI based on the KDIGO (Kidney Disease: Improving Global Outcomes) AKI classification system after any type of surgery was performed and reported outcomes using scoring systems or algorithms. Studies were also included if they reported external validation of a primary risk model. However, studies were excluded if their model was not performed clearly enough to be used in a clinical setting or if the utilized models including intraoperative or pro-operative variables, models predicting AKI requiring dialysis and models predicting AKI independent of the need for dialysis. Unpublished conference abstracts were also excluded.

All analyses were performed using IBM SPSS version 22.0 (SPSS Inc., Chicago, Illinois, USA) and SAS version 9.3 (Cary, NC). Continuous variables were presented as the mean ± standard deviation (SD) or as the median and interquartile range (IQR) when the data exhibited a skewed distribution. Analysis of variance (ANOVA) or post-hoc analysis of the least significant difference (LSD) were used to compare means between normally distributed continuous variables; otherwise, the Kruskal-Wallis test was used. Categorical variables were expressed as proportions and tested using chi-squared tests. P values of 0.05 (two-tailed) were established as the threshold for statistical significance.

## Results

### Baseline Characteristics and Outcomes

Surgery-related AKI developed in 1994 hospitalized patients (8.2%). The baseline characteristics of the study population are shown in Table 1. The mean age of the total population was 57.95±13.98 years, and 12,356 (50.5%) of the patients were male. The median length of stay in the hospital was 10 days (interquartile range, 7–17 days). The mean BMI, systolic BP, diastolic BP, and estimated glomerular filtration rate (eGFR) were 24.27±3.25 Kg/m^2^, 137.95±19.70 mmHg, 80.88±12.22 mmHg, and 77.80±32.28 mL/min/1.73 m^2^, respectively, and CKD, diabetes mellitus, heart failure, pulmonary infection, and respiratory failure were observed in 23.0%, 20.4%, 12.0%, 10.4%, and 0.6% of patients, respectively, before surgery.

**Table 1:** Baseline Characteristics of Subjects in the Training and Validation Cohorts.

	Total Cohort (n = 24451)	Training Cohort (n = 17020)	Validation Cohort (n = 7362)
Age (years)	57.95±13.98	57.92±13.97	58.03±14.00
Male sex (%)	50.5	50.5	50.6
BMI (kg/m^2^)	24.27±3.25	24.25±3.23	24.31±3.29
SBP (mmHg)	137.95±19.70	138.00±19.61	137.81±19.91
DBP (mmHg)	80.88±12.22	80.94±12.13	80.75±12.44
Duration of hypertension (years)	8.17±7.77	8.03±7.71	8.46±7.90
Current smoker (%)	20.9	21.0	20.6
Alcohol consumption (%)	12.5	12.5	12.6
eGFR	77.80±32.28	77.66±32.33	77.97±32.30
Diagnoses			
CKD (%)	23	23	22.8
Diabetes mellitus (%)	20.4	20.6	20.0
Heart failure (%)	12.0	12.1	11.9
Virus hepatitis (%)	3.5	3.5	3.6
Pulmonary infection (%)	10.4	10.3	10.6
Respiratory failure (%)	0.6	0.6	0.7
COPD (%)	2.0	2.0	2.0
Peripheral vascular disease (%)	2.6	2.5	2.7
History of disease			
Cancer	7.8	7.9	7.5
Acute coronary syndrome	4.6	4.7	4.4
Acute cerebrovascular events	7.8	7.9	7.4
Drug use			
Insulin (%)	11.1	11.1	10.9
Oral anti-diabetic drugs (%)	5.6	5.5	5.7
Contrast medium (%)	1.8	1.8	1.6
Vasodilator (%)	23.5	23.4	23.7
Antiplatelet or anticoagulant (%)	37.1	36.9	37.6
Lipid-regulating drugs (%)	15.4	15.4	15.5
Cardiotonic drugs (%)	0.4	0.4	0.4
Anti-arrhythmic drugs (%)	1.1	1.0	1.3
Anti-shock drugs (%)	5.0	4.8	5.3
Laboratory examinations			
Neutrophils (*10^9^/L)*	5.70±4.06	5.68±3.75	5.75±4.69
Lymphocytes (*10^9^/L)	1.59±0.95	1.59±0.70	1.59±1.36
NLR*	4.53±4.40	4.51±4.41	4.58±4.38
Eosinophils (*10^9^/L)	0.14±0.17	0.14±0.17	0.14±0.19
Basophils (*10^9^/L)	0.02±0.10	0.02±0.10	0.02±0.10
Red blood cells (*10^12^/L)	4.12±0.78	4.12±0.78	4.13±0.78
Hemoglobin (g/L)	121.54±23.65	121.35±23.70	121.96±23.53
Blood platelets (*10^9^/L)*	194.78±71.14	195.58±71.80	192.92±69.54
Glucose (mmol/L)*	5.76±2.23	5.77±2.29	5.74±2.09
ALT (u/L)	29.43±74.91	29.53±83.25	29.20±50.33
AST (u/L)	32.23±91.18	32.46±103.15	31.68±83.25
Serum total protein (g/L)	66.31±7.65	66.29±7.66	66.36±7.62
Serum albumin (g/L)	39.37±5.31	39.37±5.31	39.40±5.27
Serum globulin (g/L)	26.94±4.85	26.93±4.88	26.96±4.77
Total cholesterol (mmol/L)	4.67±1.20	4.67±1.20	4.69±1.89
LDL (mmol/L)	2.58±0.89	2.60±0.89	2.57±0.90
HDL (mmol/L)	0.79±0.23	0.79±0.23	0.79±0.24
Uric acid (μmol/L)	321.25±111.90	321.05±112.05	321.71±111.33
BUN (mmol/L)	7.16±5.90	7.14±5.87	7.19±5.97
TT (s)	15.76±7.85	15.75±7.69	15.79±8.23
PT (s)	11.80±3.00	11.80±3.17	11.79±2.56
INR	1.01±0.20	1.01±0.20	1.01±0.20
Serum potassium (mmol/L)	4.07±0.48	4.07±0.48	4.07±0.49
Serum sodium (mmol/L)	140.37±3.08	140.36±3.07	140.39±3.10
Serum chlorine (mmol/L)	104.39±3.76	104.38±3.78	104.42±3.10
Serum calcium (mmol/L)	2.26±0.18	2.26±0.18	2.26±0.18
CO_2_CP (mmol/l)	23.92±3.73	23.91±3.73	23.95±3.74
Anion gap (mmol/l)	16.42±3.97	16.42±3.94	16.41±4.04
Urine pH	6.23±0.71	6.23±0.71	6.23±0.72
Urine specific gravity	1.02±0.07	1.02±0.01	1.02±0.01
Surgical sites			
EENT surgery (%)	9.5	9.4	9.6
Blood and lymphatic system surgery (%)	1.6	1.6	1.6
Cardiovascular surgery (%)	12.8	12.7	13.0
Digestive surgery (%)	19.8	20.0	19.3
Integumentary system surgery (%)	3.1	3.0	3.2
Nervous system surgery (%)	4.3	4.0	4.9
Urogenital surgery (%)	26.1	26.1	25.9
Endocrine surgery (%)	2.7	2.7	2.7
Musculoskeletal surgery (%)	6.5	6.4	6.6
Respiratory surgery (%)	3.2	3.3	3.1
Other sites (%)	10.6	10.9	10.2

Data are numbers (percentage) and mean ± standard deviation. ALT, glutamic oxaloacetic transaminase; AST, aspartate amino transferase; BMI, body mass index; BUN, blood urea nitrogen; CKD, chronic kidney disease; CO_2_CP, carbon dioxide combining power; COPD, chronic obstructive pulmonary disease; DBP, diastolic blood pressure; EENT, ear, eye, nose and throat surgery; eGFR, estimated glomerular filtration rate; HDL, high-density lipoprotein cholesterol; INR, International Normalized Ratio; LDL, low-density lipoprotein cholesterol; NLR, neutrophil-to-lymphocyte ratio; PT, prothrombin time; PVD, peripheral vascular disease; SBP, systolic blood pressure, TT, thrombin time.

*For the difference between patients in the Training Cohort and patients in the Validation Cohort, *P* value<0.05.

### AKI Predictors and Risk Model Development

Sixty-six potential variables were evaluated in the training set used for model inclusion, and variable significance scores were computed using Fisher’s linear discriminant analysis method. The top 31 variables with weights greater than 0.99 were selected (S1 Table). eGFR had the highest score. Hematocrit (VIF = 11.74), total protein (VIF = 103.37), globulin (VIF = 72.07), serum calcium (VIF = 26.07), serum sodium (VIF = 202.11), serum chloride (VIF = 163.73) and INR (VIF = 9.48) were deleted for multicollinearity purposes when constructing different models. The potential predictive models for surgery-related AKI were then sequentially developed using stepwise multivariate logistic regression, and their AUCs and AIC were calculated (S2 Table). The inclusion of additional variables did not significantly improve prediction efficiency. Therefore, our predictive model, known as the Xiang-ya model, was model 13 and comprised the following thirteen variables (Table 2): age, gender, eGFR, NLR, pulmonary infection, prothrombin time (PT), thrombin time (TT), hemoglobin, uric acid, serum potassium, serum albumin, total cholesterol, and aspartate amino transferase (AST). The AUC and AIC were 0.89 (0.88–0.90) and 6515.93, respectively, with respect to surgery-related AKI prediction.

**Table 2:** Xiany-ya Risk Model Calculation Formula.

Variables	Coefficient
eGFR	-0.034
NLR	0.067
TT	0.015
Serum potassium	0.259
Pulmonary infection (1 if present)	0.514
Age	-0.016
Uric acid	-0.002
Serum albumin	-0.020
AST	0.002
Total cholesterol	0.071
Gender (1 if male, 2 if female)	-0.314
PT	0.021
Hemoglobin	-0.006

The probability of significant surgery-associated AKI was calculated as

1/(1 + *e*^−*x*^)

where *e* = base of the natural logarithm, where *x* = *a*_*1*_*y*_*1*_*+a*_*2*_*y*_*2*_*+…+a*_*k*_*y*_*k*_*+B*, where y_1_, y_2_, …, y_k_ are the characteristics, where a_1_, a_2_, …, a_k_ are the corresponding logistic regression coefficients, and where B is the intercept term (in this case, 0.694).

The predictive characteristics are listed here with their coefficients.

AST, aspartate amino transferase; BUN, blood urea nitrogen; eGFR, estimated glomerular filtration rate; NLR, neutrophil-to-lymphocyte ratio; PT, prothrombin time; TT, thrombin time

### Risk Model Validation

**Fig 1:**
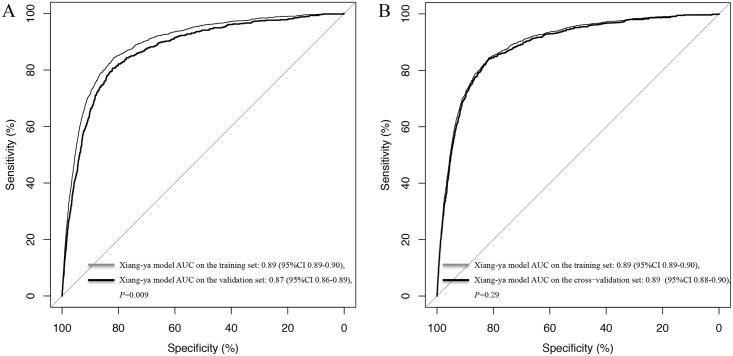
Receiver Operating Characteristic Curves for the Xiang-ya Model of Surgery-related AKI in the Training and Validation Sets (A) and in the Training and Cross-Validation Set (B). AUC, area under the receiver operating characteristic curve.

The validation set consisted of 7362 hypertensive patients who underwent surgery, 598 of whom developed AKI. The AUC for the validation set was 0.87 (95% CI 0.86–0.89), which was similar to the AUC for the training set (0.89; 95% CI 0.88–0.90) (Fig 1). Cross validation exhibited a significant predictive accuracy for AKI (AUC = 0.89, 95% CI 0.88–0.90), and training error and validation error were both 0.08, indicating that there was no over-fitting in our model. The predictive power of the Xiang-ya model in the training set at different cutoff values is listed in Table 3. The optimal cutoff value was 0.09, which predicted surgery-related AKI with a sensitivity of 79% and a specificity of 86%.

**Table 3:** Diagnostic and Predictive Values of the Xiang-ya Risk Model at Different Cutoff Points for Predicted Probability.

Cutoff point for predicted probability	Sensitivity	Specificity	Positive predictive value	Negative predicted value	Youden index	G_mean_
0.05	0.87	0.77	0.26	0.98	0.64	0.82
0.06	0.85	0.81	0.29	0.98	0.66	0.83
0.07	0.82	0.83	0.31	0.98	0.65	0.83
0.08	0.8	0.85	0.32	0.98	0.65	0.83
0.09	0.79	0.86	0.34	0.98	0.65	0.83
0.10	0.77	0.87	0.36	0.98	0.64	0.82
0.15	0.71	0.9	0.4	0.97	0.61	0.8
0.20	0.67	0.92	0.43	0.97	0.59	0.78
0.30	0.56	0.94	0.47	0.96	0.51	0.73

### Comparison with Other AKI Risk Models

**Fig 2:**
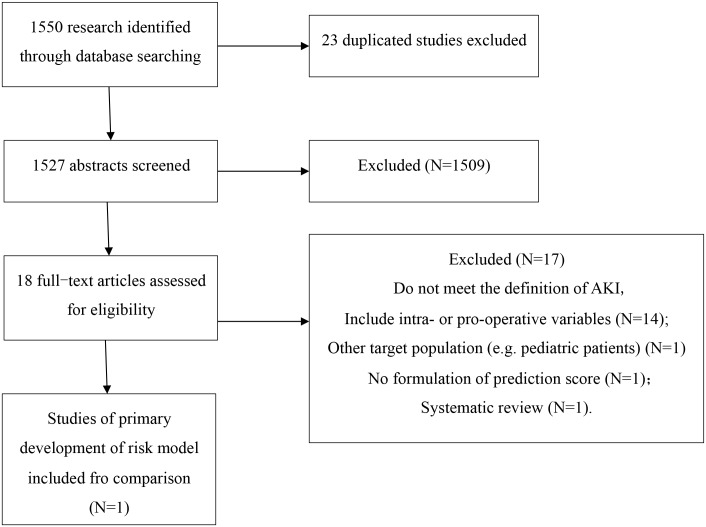
Flow Chart of Eligible Studies for Model Comparison.

**Fig 3:**
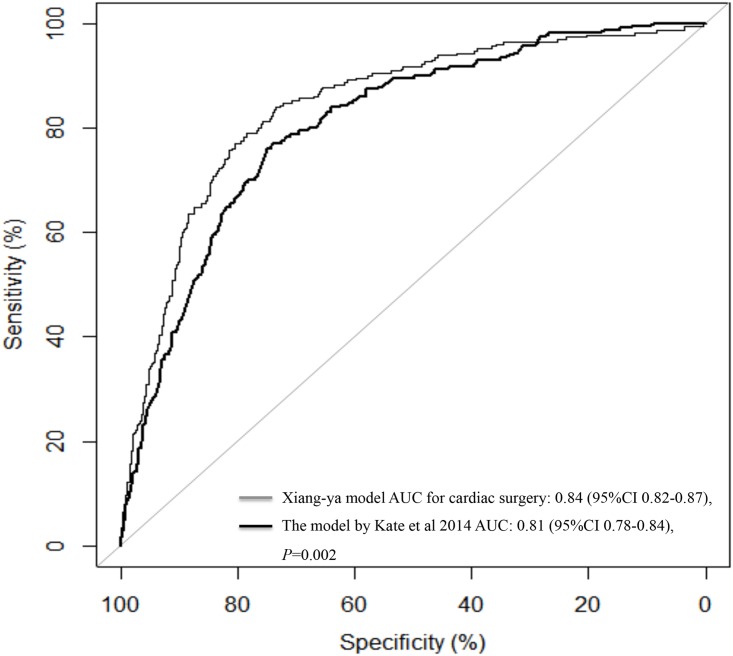
Comparison of the Receiver Operating Characteristic Curves for the Xiang-ya Model and the Model Proposed by Kate et al 2014 for Predicting Cardiac Surgery-related AKI (n = 2101). AUC, area under the receiver operating characteristic curve.

A total of 1550 studies were identified and screened, and we identified 18 relevant articles [15–31] containing information regarding prediction scores or algorithms pertaining to surgery-related AKI. However, only the model developed by Kate et al [19], which produced a risk score to predict AKI after cardiac surgery in a cohort of 30,854 patients from 3 UK cardiac surgical centers, fulfilled our inclusion criteria (Fig 2). Our model (AUC = 0.84, 95% CI 0.82–0.87) demonstrated better discriminatory power than the model developed by Kate et al (AUC = 0.81, 95% CI 0.78–0.84) with respect to predicting cardiac surgery-related AKI (Fig 3). Our model also outperformed the model developed by Kate et al when assessed using the net reclassification index and integrated discrimination improvement, exhibiting an NRI of 31.38% (95% CI 25.7%-37.1%) and an IDI of 8% (95% CI 5.52%-10.50%) in patients who underwent cardiac surgery (n = 2101).

## Discussion

AKI is a serious and frequent post-surgery complication, and many factors are associated with the risk of AKI development. A common feature of a larger number of the processes causing AKI is a reduction in regional renal oxygen delivery, leading to inflammation, ischemia and necrosis [32]. Hypertension remains a powerful prognostic marker for this condition [11,33], for which no specific therapeutic regimens exist [34,35]. The etiology of hypertension in renal damage involves mechanisms with inter-related complications, such as balance disorders of vascular active substances, RAS activation, changes in inflammatory factors, and increases in active oxygen species [36]. Inflammation has been implicated in the pathophysiology of hypertension via both changes in endothelial function and arterial stiffness [37], with reduced availability of nitric oxide (NO) being integral to this process [38]. Oxidative stress also appears to be an important feature in the reduced availability of NO and is aggravated by an increase in circulating angiotensin II (Ang II) levels. The combination of hypertension, increased angiotensin II levels and oxidative stress initiates events leading to renal damage [39].

Although the addition of intra- and early postoperative information substantially improved our ability to predict AKI, preoperative risk stratification tools would allow the identification of high-risk patients and enable prevention strategies to be implemented earlier, thereby improving quality of care, patient management, and resource allocation. Hence, it is essential to evaluate and predict AKI before surgery in hypertensive patients.

We selected and analyzed 24,451 hypertensive patients to develop a risk prediction model for surgery-related AKI. The main findings of this study are as follows: (1) hypertensive patients who underwent general surgery exhibited a high incidence of postoperative AKI (8.2%); (2) preoperative renal function was the factor most strongly associated with AKI; and (3) our model, which was based on thirteen preoperative risk factors and biomarkers, feasibly predicted AKI in hypertensive patients who underwent general surgery.

One strength of our study was that AKI events could be detected using the structured HIS of the 3^rd^ Xiangya Hospital, which serves as the sole portal for clinical data entry for all public inpatient settings across different regions of China and provides quality-controlled and reliable data. Another strength of our study was that it focused on all types of operations and nonetheless achieved better discriminatory ability in patients undergoing cardiac surgery. Most other AKI risk prediction models have not been adequately tested in this population. Furthermore, we applied a well-established KDIGO definition to enhance the applicability of this model for other studies, as well as the comparability of its data with those from other studies. We identified only one existing risk prediction tool [25] that predicted general surgery-related AKI. However, AKI was defined as “progressive renal insufficiency or acute renal failure necessitating dialysis within 30 days after surgery” in that study. Several different AKI definitions have been used to develop risk models, including definitions mentioning requirements for dialysis and eGFRs <30 mL/min/1.73 m^2^, as well as other definitions based on various absolute or relative increases in serum creatinine concentrations.

The important covariates in our prediction model were age, gender, eGFR, NLR, pulmonary infection, PT, TT, hemoglobin, uric acid, serum potassium, serum albumin, total cholesterol, and AST. To increase the predictive ability of our model, we adopted continuous variables as often as possible rather than defining cutoffs. The incorporated data indicated that the overall renal function and the general condition (male, anemia, hypo-albuminemia, prolonged TT, PT, elevation of liver transaminase, and hyperkalemia) of the patient were predictive of AKI events. It is not surprising that age and hypercholesterolemia were risk factors identified in our model because they have a remarkable effect on the arterial system and markedly increases the risk of cardiovascular events, such as atherosclerosis, stroke, and myocardial infarction. In addition, the NLR (an inflammatory marker) and preoperative pulmonary infection were independently significantly associated with AKI in our model. These variables are considered markers of sepsis, which is the leading cause of AKI in clinical practice, and can be controlled before elective surgery [40,41]. These results are consistent with those of previous studies demonstrating that elevated preoperative neutrophil counts or NLRs are consistently found to be significant independent risk factors for AKI development [41,42]. However, surgical site was not associated with AKI in our model, perhaps because surgical site did not affect other variables, including operation trauma, operation time, intraoperative complications, or anesthesia type.

The optimal cutoff value was 0.09, indicating that 34.29% (positive predictive value) of participants with a score >0.09 were diagnosed with AKI, whereas 97.84% (negative predictive value) of participants with a score <0.09 were not diagnosed with AKI. From a clinical point of view, however, the cutoff value should be chosen according to prevention strategies.

The potential limitations of our study should be mentioned. First, this study was conducted in a single center and may therefore be weaker in methodology than studies using randomly sampled populations. Second, the patients in our study who developed AKI were not classified according to AKI severity (e.g., AKI 1, AKI 2, and AKI 3). Third, although predictable intraoperative variables (e.g., operation time, operation trauma, anesthesia type) and novel biomarkers, such as microRNAs [43], urine neutrophil gelatinase-associated lipocalin (NGAL) [44–47], cystatin C [44], fibroblast growth factor-23 [48], and interleukin-18 [46,49], have been proposed as alternative methods for detecting individuals at high risk for AKI, the analysis used in the present study was limited to measurements of variables available in <5% of study participants. Moreover, although combining these specific biomarkers may improve predictive model effectiveness, it is not financially feasible to screen every patient. Fourth, Xiang-ya risk model may not be generalizable to all kinds of hypertensive patients (e.g. hypertension and CKD patients)(S1 Fig). Preoperative renal function is the most important risk factor of surgery-related AKI. Unlike hypertension, CKD can occur through diverse pathologic mechanisms injuring several compartments of the kidney (i.e., the vasculature, the tubulointerstitium and/or the glomerulus) [50]. According to a recent meta-analysis, when eGFRs>60 mL/min/1.73 m^2^, hypertensive patients had a higher risk of AKI at than those without hypertension. However, hypertension has little impact on the risk of AKI when eGFRs<60 mL/min/1.73 m^2^ [33]. Therefore, the risk model of surgery-related AKI must be different in hypertensive patients and CKD patients. Moreover, to achieve a good model performance, we attempted to find rules and train a model based on large amounts of data. However, data pertaining to these patients are limited; therefore, the prediction performance for some specific HTN patients was less effective than for general HTN patients. Finally, although our study developed a feasible risk prediction model, such models remain seldom used in clinical practice. Future studies addressing these limitations are necessary, and a web-based or HIS integration calculator for this prediction should also be developed so that this model is accessible to clinicians.

## Conclusions

In summary, we developed an AKI risk model based on preoperative risk factors and biomarkers and demonstrated that it provides an acceptable level of performance for predicting events in a large cohort of hypertensive patients who underwent general surgery. However, the applicability of this proposed risk model should be evaluated in additional studies.

## Supporting Information

S1 Fig Receiver Operating Characteristic Curves for the Xiang-ya Model of Surgery-related AKI in the HTN patients and HTN complicated with CKD (A), HTN complicated with DM (B), HTN complicated with HF (C), and HTN with complicated PVD (D) patients. CKD, chronic kidney disease; DM, diabetes mellitus; HF, heart failure; HTN, hypertension; PVD, peripheral vascular disease.
(EPS)

S1 Table Importance Scores for the Variables Used to Determine AKI Risk, as Determined Using Fisher’s Linear Discriminant Analysis Method. (DOCX)

S2 Table Performance of the Xiany-ya Risk Model Using Stepwise Multivariate Logistic Regression in the Training Cohort. (DOCX)

S1 Text Scientific Research Project Approval. (PDF)

S2 Text Data Security Confidentiality Agreement for the Xiang-ya Big Data Project of the Third Xiangya Hospital, Central South University. (PDF)
