# Supplementary material for: A Predictive Model for Assessing Surgery-Related Acute Kidney Injury Risk in Hypertensive Patients: A Retrospective Cohort Study
Source: PLoS One. 2016 Nov 1;11(11):e0165280. doi: 10.1371/journal.pone.0165280 (PMC5089779; doi:10.1371/journal.pone.0165280)
Supplement: S1 Table — (DOCX) [file pone.0165280.s002.docx]

**S1 Table. Importance Scores for the Variables Used to Determine AKI Risk, as Determined Using Fisher’s Linear Discriminant Analysis Method**

| Variables | AKI | |
| --- | --- | --- |
|  | Importance Score | Relative Importance Score |
| eGFR | 0.204 | 0.177 |
| BUN | 0.194 | 0.168 |
| Hematocrit | 0.100 | 0.087 |
| CKD | 0.100 | 0.087 |
| Red blood cells | 0.095 | 0.083 |
| Hemoglobin | 0.093 | 0.081 |
| Serum albumin | 0.045 | 0.039 |
| Serum total protein | 0.042 | 0.036 |
| NLR | 0.040 | 0.034 |
| Serum potassium | 0.037 | 0.032 |
| Serum calcium | 0.036 | 0.031 |
| Pulmonary infection | 0.027 | 0.023 |
| INR | 0.014 | 0.012 |
| Uric acid | 0.014 | 0.012 |
| PT | 0.010 | 0.009 |
| Age | 0.010 | 0.009 |
| Serum sodium | 0.010 | 0.009 |
| Total cholesterol | 0.010 | 0.009 |
| Heart Failure | 0.008 | 0.007 |
| Serum globulin | 0.008 | 0.007 |
| Gender | 0.006 | 0.005 |
| Platelet | 0.006 | 0.005 |
| Thrombin time | 0.005 | 0.005 |
| Specific gravity of urine | 0.005 | 0.004 |
| AST | 0.005 | 0.004 |
| Blood chloride | 0.004 | 0.004 |
| Respiratory failure | 0.004 | 0.004 |
| Virus hepatitis | 0.003 | 0.002 |
| MPV | 0.002 | 0.002 |
| PVD | 0.002 | 0.002 |

AST: Aspartate amino transferase; BUN, blood urea nitrogen; CKD, chronic kidney disease; eGFR, estimated glomerular filtration rate; INR: International Normalized Ratio; MPV: mean platelet volume; NLR, neutrophil to lymphocyte ratio; PVD, Peripheral vascular disease; PT, prothrombin time; TT, thrombin time.
