# Supplementary material for: A Predictive Model for Assessing Surgery-Related Acute Kidney Injury Risk in Hypertensive Patients: A Retrospective Cohort Study
Source: PLoS One. 2016 Nov 1;11(11):e0165280. doi: 10.1371/journal.pone.0165280 (PMC5089779; doi:10.1371/journal.pone.0165280)
Supplement: S2 Table — (DOCX) [file pone.0165280.s003.docx]

**S2 Table. Performance of the Xiany-ya Risk Model Using Stepwise Multivariate Logistic Regression in the Training Cohort**

| Models | Variables | Coefficient | Odd Ratio | P Valve |
| --- | --- | --- | --- | --- |
| 1 | eGFR | -0.042 | 0.959 | <0.001 |
|  | AUC (95% confidence interval) |  | 0.849(0.837-0.861) |  |
|  | AIC |  | 7086.848 |  |
|  | Likelihood ratio |  | 2585.342 |  |
| 2 | eGFR | -0.041 | 0.960 | <0.001 |
|  | NLR | 0.084 | 1.087 | <0.001 |
|  | AUC (95% confidence interval) |  | 0.869(0.859-0.880) |  |
|  | AIC |  | 6790.339 |  |
|  | Likelihood ratio |  | 2790.127 |  |
| 3 | eGFR | -0.041 | 0.960 | <0.001 |
|  | NLR | 0.081 | 1.084 | <0.001 |
|  | TT | 0.024 | 1.024 | <0.001 |
|  | AUC (95% confidence interval) |  | 0.874(0.864-0.884) |  |
|  | AIC |  | 6719.597 |  |
|  | Likelihood ratio |  | 2862.869 |  |
| 4 | eGFR | -0.038 | 0.963 | <0.001 |
|  | NLR | 0.083 | 1.086 | <0.001 |
|  | TT | 0.023 | 1.023 | <0.001 |
|  | Serum potassium | 0.285 | 1.330 | <0.001 |
|  | AUC (95% confidence interval) |  | 0.880 (0.870-0.889) |  |
|  | AIC |  | 6653.388 |  |
|  | Likelihood ratio |  | 2931.078 |  |
| 5 | eGFR | -0.037 | 0.963 | <0.001 |
|  | NLR | 0.075 | 1.078 | <0.001 |
|  | TT | 0.023 | 1.023 | <0.001 |
|  | Serum potassium | 0.285 | 1.329 | <0.001 |
|  | Pulmonary infection | 0.558 | 1.748 | <0.001 |
|  | AUC (95% confidence interval) |  | 0.882 (0.872-0.892) |  |
|  | AIC |  | 6609.046 |  |
|  | Likelihood ratio |  | 2977.420 |  |
| 6 | eGFR | -0.035 | 0.965 | <0.001 |
|  | NLR | 0.077 | 1.08 | <0.001 |
|  | TT | 0.023 | 1.023 | <0.001 |
|  | Serum potassium | 0.276 | 1.318 | <0.001 |
|  | Pulmonary infection | 0.618 | 1.855 | <0.001 |
|  | Age | -0.014 | 0.986 | <0.001 |
|  | AUC (95% confidence interval) |  | 0.885 (0.876-0.895） |  |
|  | AIC |  | 6567.677 |  |
|  | Likelihood ratio |  | 3020.789 |  |
| 7 | eGFR | -0.038 | 0.963 | <0.001 |
|  | NLR | 0.074 | 1.077 | <0.001 |
|  | TT | 0.022 | 1.023 | <0.001 |
|  | Serum potassium | 0.272 | 1.313 | <0.001 |
|  | Pulmonary infection | 0.614 | 1.848 | <0.001 |
|  | Age | -0.014 | 0.986 | <0.001 |
|  | Uric acid | -0.002 | 0.998 | <0.001 |
|  | AUC (95% confidence interval) |  | 0.886 (0.877-0.896) |  |
|  | AIC |  | 6535.773 |  |
|  | Likelihood ratio |  | 3054.6932 |  |
| 8 | eGFR | -0.037 | 0.964 | <0.001 |
|  | NLR | 0.069 | 1.072 | <0.001 |
|  | TT | 0.022 | 1.023 | <0.001 |
|  | Serum potassium | 0.280 | 1.324 | <0.001 |
|  | Pulmonary infection | 0.576 | 1.778 | <0.001 |
|  | Age | -0.015 | 0.985 | <0.001 |
|  | Uric acid | -0.002 | 0.998 | <0.001 |
|  | Serum albumin | -0.021 | 0.979 | <0.001 |
|  | AUC (95% confidence interval) |  | 0.889 (0.880-0.898) |  |
|  | AIC |  | 6515.925 |  |
|  | Likelihood ratio |  | 3076.5406 |  |
| 9 | eGFR | -0.037 | 0.964 | <0.001 |
|  | NLR | 0.067 | 1.069 | <0.001 |
|  | TT | 0.021 | 1.021 | <0.001 |
|  | Serum potassium | 0.281 | 1.324 | <0.001 |
|  | Pulmonary infection | 0.558 | 1.747 | <0.001 |
|  | Age | -0.015 | 0.985 | <0.001 |
|  | Uric acid | -0.002 | 0.998 | <0.001 |
|  | Serum albumin | -0.021 | 0.979 | <0.001 |
|  | AST | 0.002 | 1.002 | <0.001 |
|  | AUC (95% confidence interval) |  | 0.891 (0.882-0.900) |  |
|  | AIC |  | 6495.371 |  |
|  | Likelihood ratio |  | 3099.0950 |  |
| 10 | eGFR | -0.037 | 0.963 | <0.001 |
|  | NLR | 0.069 | 1.072 | <0.001 |
|  | TT | 0.020 | 1.021 | <0.001 |
|  | Serum potassium | 0.269 | 1.309 | <0.001 |
|  | Pulmonary infection | 0.544 | 1.722 | <0.001 |
|  | Age | -0.016 | 0.984 | <0.001 |
|  | Uric acid | -0.002 | 0.998 | <0.001 |
|  | Serum albumin | -0.023 | 0.977 | <0.001 |
|  | AST | 0.002 | 1.002 | <0.001 |
|  | Total cholesterol | 0.057 | 1.058 | 0.0003 |
|  | AUC (95% confidence interval) |  | 0.891 (0.883-0.900) |  |
|  | AIC |  | 6484.421 |  |
|  | Likelihood ratio |  | 3112.044 |  |
| 11 | eGFR | -0.037 | 0.963 | <0.001 |
|  | NLR | 0.069 | 1.071 | <0.001 |
|  | TT | 0.020 | 1.02 | <0.001 |
|  | Serum potassium | 0.262 | 1.299 | <0.001 |
|  | Pulmonary infection | 0.534 | 1.705 | <0.001 |
|  | Age | -0.016 | 0.984 | <0.001 |
|  | Uric acid | -0.002 | 0.998 | <0.001 |
|  | Serum albumin | -0.024 | 0.976 | <0.001 |
|  | AST | 0.002 | 1.002 | <0.001 |
|  | Total cholesterol | 0.063 | 1.065 | <0.001 |
|  | Gender | -0.261 | 0.77 | 0.0002 |
|  | AUC (95% confidence interval) |  | 0.892 (0.883-0.901) |  |
|  | AIC |  | 6472.201 |  |
|  | Likelihood ratio |  | 3126.265 |  |
| 12 | eGFR | -0.037 | 0.964 | <0.001 |
|  | NLR | 0.067 | 1.069 | <0.001 |
|  | TT | 0.015 | 1.015 | <0.001 |
|  | Serum potassium | 0.266 | 1.304 | <0.001 |
|  | Pulmonary infection | 0.517 | 1.676 | <0.001 |
|  | Age | -0.017 | 0.984 | <0.001 |
|  | Uric acid | -0.002 | 0.998 | <0.001 |
|  | Serum albumin | -0.024 | 0.976 | <0.001 |
|  | AST | 0.002 | 1.002 | <0.001 |
|  | Total cholesterol | 0.067 | 1.069 | <0.001 |
|  | Gender | -0.260 | 0.771 | 0.0002 |
|  | PT | 0.022 | 1.022 | 0.0009 |
|  | AUC (95% confidence interval) |  | 0.893 (0.884-0.902) |  |
|  | AIC |  | 6462.093 |  |
|  | Likelihood ratio |  | 3138.373 |  |
| 13 | eGFR | -0.034 | 0.966 | <0.001 |
|  | NLR | 0.067 | 1.069 | <0.001 |
|  | TT | 0.015 | 1.015 | <0.001 |
|  | Serum potassium | 0.259 | 1.296 | <0.001 |
|  | Pulmonary infection | 0.514 | 1.672 | <0.001 |
|  | Age | -0.016 | 0.985 | <0.001 |
|  | Uric acid | -0.002 | 0.998 | <0.001 |
|  | Serum albumin | -0.020 | 0.981 | <0.001 |
|  | AST | 0.002 | 1.002 | <0.001 |
|  | Total cholesterol | 0.071 | 1.073 | <0.001 |
|  | Gender | -0.314 | 0.731 | <0.001 |
|  | PT | 0.021 | 1.021 | 0.0014 |
|  | Hemoglobin | -0.006 | 0.995 | 0.0009 |
|  | AUC (95% confidence interval) |  | 0.894 (0.885-0.903) |  |
|  | AIC |  | 6453.110 |  |
|  | Likelihood ratio |  | 3149.356 |  |
| 14 | eGFR | -0.034 | 0.966 | <0.001 |
|  | NLR | 0.067 | 1.069 | <0.001 |
|  | TT | 0.015 | 1.015 | <0.001 |
|  | Serum potassium | 0.255 | 1.29 | <0.001 |
|  | Pulmonary infection | 0.507 | 1.66 | <0.001 |
|  | Age | -0.016 | 0.985 | <0.001 |
|  | Uric acid | -0.002 | 0.998 | <0.001 |
|  | Serum albumin | -0.020 | 0.981 | <0.001 |
|  | AST | 0.002 | 1.002 | <0.001 |
|  | Total cholesterol | 0.069 | 1.071 | <0.001 |
|  | Gender | -0.314 | 0.73 | <0.001 |
|  | PT | 0.021 | 1.022 | 0.001 |
|  | Hemoglobin | -0.005 | 0.995 | 0.001 |
|  | PVD | 0.551 | 1.735 | 0.0011 |
|  | AUC (95% confidence interval) |  | 0.894 (0.885-0.903) |  |
|  | AIC |  | 6444.997 |  |
|  | Likelihood ratio |  | 3159.468 |  |

AST, aspartate amino transferase; BUN, blood urea nitrogen; eGFR, estimated glomerular filtration rate; NLR, neutrophil-to-lymphocyte ratio; PVD, peripheral vascular disease; PT, prothrombin time; TT, thrombin time
