# Supplementary material for: A Predictive Model for Assessing Surgery-Related Acute Kidney Injury Risk in Hypertensive Patients: A Retrospective Cohort Study
Source: PLoS One. 2016 Nov 1;11(11):e0165280. doi: 10.1371/journal.pone.0165280 (PMC5089779; doi:10.1371/journal.pone.0165280)
Supplement: S1 Text — (PDF) [file pone.0165280.s004.pdf]

中南大學湘雅三医院
The Third Xiangya Hospital of Central South University

科学研究项目审批件

Approval for Scientific Research Project

No: 2016-149

<table>
<tr><td>项目名称
Project Title</td><td colspan="5">高血压患者手术相关急性肾损伤的风险评估模型
Model for Assessing surgery related AKI in hypertension patients</td></tr>
<tr><td>专业科室
Department</td><td>心内科/临床药理中心
Cardiology/Clinical Pharmacology Center</td><td>主要研究者
Principal Investigator</td><td>袁洪
Hong Yuan</td><td>承担责任
Position</td><td>负责 In charge ☑
参与 Participants ☐</td></tr>
<tr><td>研究分类
Classification</td><td colspan="5">1 病理标本研究 Pathology specimens research ☐
2 人体试验 Human research ☑
3 人体组织或细胞实验 Human tissue or cell experiments ☐
4 胚胎克隆研究 Research of embryonic clone ☐
5 其他研究 Other research ☐</td></tr>
<tr><td>审查意见
Conclusion</td><td colspan="5">同意 Agree ☑； 作必要修正后同意 Agree after necessary revisions☐；
不同意 Disagree☐；终止或暂停已批准的试验 Termination or suspension of the approved research☐</td></tr>
<tr><td>审查声明
Statement</td><td colspan="5">兹证明本研究的设计和方法符合相关法规（GCP、ICH-GCP 等）和伦理原则的要求。伦理委员会批准本研究项目在本院执行。
This is to certify that the design and methods of the research are in accordance with the requirements of related regulations and procedures (such as GCP, ICH-GCP) as well as the ethical principles. The IRB has approved the research to be conducted in our hospital.

中南大学湘雅三医院伦理委员会医学伦理分委员会
The IRB of Third Xiangya Hospital, Central South University
2016 年 4 月 25 日</td></tr>
</table>
