# Supplementary material for: A Predictive Model for Assessing Surgery-Related Acute Kidney Injury Risk in Hypertensive Patients: A Retrospective Cohort Study
Source: PLoS One. 2016 Nov 1;11(11):e0165280. doi: 10.1371/journal.pone.0165280 (PMC5089779; doi:10.1371/journal.pone.0165280)
Supplement: S2 Text — (PDF) [file pone.0165280.s005.pdf]

中南大学湘雅三医院大数据项目
数据安全保密协议

Data Security Confidentiality Agreement for the Xiang-ya Big Data Project of the Third Xiangya Hospital, Central South University

鉴于中南大学湘雅三医院大数据项目的启动，为了更好的发挥信息数据在今后临床实践及教研中的功能和作用，同时也保障医院信息数据的稳定与安全，为相关工作的开展提供有力支撑，经院务会讨论通过，参与大数据项目的相关课题组需遵守本协议所列条款。

With the start of the Xiang-ya Big Data Project of the 3rd Xiangya Hospital of Central South University, the following requirements have been implemented by the hospital administrative committee to ensure proper use of the information obtained by the project in future clinical practice and scientific research, to ensure the stability and security of hospital information, and to support the development of related works. These requirements must be followed by all research groups involved in the project.

第一、中南大学湘雅三医院大数据项目的全部医院数据均保存在信息中心，不允许采取外接设备拷贝离院。

1. All data pertaining to the Xiang-ya Big Data Project of the 3rd Xiang-ya Hospital, Central South University, must be physically stored in the Information Department, the Third Xiang-ya Hospital, Central South University, from where these data cannot be copied by external devices.

第二、大数据项目中涉及患者多种信息，项目负责人需确保

Add:138,Tongzipo Road,Changsha,Hunan(410013),China Tel:86-731-88638888 Fax:86-731-88921910 Website:www.xy3yy.com

隐私数据的安全，不允许采集相关隐私数据。

2. The principal investigators must ensure the security of private patient data.

第三、大数据项目成果和产品将由医院、项目负责人、第三方合作单位共同拥有。

3. All achievements by and products of the Xiang-ya Big Data Project must be shared by the 3rd Xiang-ya Hospital, the principal investigators and the cooperation units.

第四、不在涉密计算机上联接和使用非涉密移动存储设备；不在非涉密计算机上联接和使用涉密移动存储设备。

4. Covert use of removable storage devices or computers is prohibited.

第五、所有项目组的数据必须履行安全保密协议，若有违反将追究责任，严重影响的将通过法律途径解决。

5. All Xiang-ya Big Data Project teams must comply with this confidentiality agreement. In the event that the agreement is broken, the 3rd Xiang-ya Hospital may pursue legal action.

如果您有任何问题，您可以在国家法定工作日的工作时间联系以下单位：

中南大学湘雅三医院科研科（+86-0731-88618470）

Please contact the following unit during national legal working hours if you have any questions: the Research Department of the 3rd Xiang-ya Hospital (+86-0731-88618470).

中南大学湘雅三医院科研部

Add:138,Tongzipo Road,Changsha,Hunan(410013),China Tel:86-731-88638888 Fax:86-731-88921910 Website:www.xy3yy.com
